# Immune evasion by a chytrid fungus includes inhibition of macrophage phagocytosis

**DOI:** 10.1128/iai.00591-25

**Published:** 2026-03-31

**Authors:** Louise A. Rollins-Smith, Mitchell Le Sage, Chelsea Roach Simoneau, Somya Singh, Muhammad Riadul Haque Hossainey, Carlos Henrique Serezani, Leon Grayfer

**Affiliations:** 1 Department of Pathology, Microbiology and Immunology, Vanderbilt University School of Medicine 12327 , Nashville, Tennessee, USA; 2 Department of Pediatrics, Vanderbilt University School of Medicine 12327 , Nashville, Tennessee, USA; 3 Department of Biological Sciences, Vanderbilt University 5718, Nashville, Tennessee, USA; 4 Department of Biological Sciences, The George Washington University 8367, Washington, DC, USA; 5 Department of Medicine, Division of Infectious Diseases, Vanderbilt University Medical Center 12328, Nashville, Tennessee, USA; 6 Vanderbilt Center for Immunobiology, Vanderbilt University Medical Center 12328, Nashville, Tennessee, USA; 7 Vanderbilt Institute of Infection, Immunology and Inflammation (VI4), Vanderbilt University Medical Center 12328, Nashville, Tennessee, USA; Tsinghua University, Beijing, China

**Keywords:** amphibian declines, *Batrachochytrium dendrobatidis*, chytrid fungus, immune defenses, immune evasion, macrophages, phagocytosis

## Abstract

*Batrachochytrium dendrobatidis* continues to cause declines in amphibian populations worldwide, and it remains unclear why skin defenses often fail to control the infection. Although amphibians have a complex, multifunctional immune system, the chytridiomycosis agent seems to have evolved countermeasures that enable it to survive and eventually impair critical skin functions. Previous studies show that *B. dendrobatidis* cells or cell-free supernatants inhibit lymphocytes by inducing apoptosis, suggesting impaired local cell killing. However, there is little evidence of lymphocyte recruitment to chytrid-infected skin, implying the fungus may also inhibit the functions of antigen-presenting cells. Here, we demonstrate that phagocytosis by peritoneal macrophages is significantly reduced by co-culture with live or heat-killed *B. dendrobatidis* zoosporangia, freeze-thawed zoospores, fungal cell-free supernatants, or cell-wall fragments. The phagocytic capacity of frog bone marrow-derived macrophages, differentiated by colony-stimulating factor-1 (CSF-1) or interleukin-34 (IL-34) (key macrophage growth factors), as well as immortalized mammalian macrophages, is also impaired. Inhibition of mammalian macrophages suggests that these inhibitory factors are not restricted to amphibian cells. Overall, these studies indicate that *B. dendrobatidis* cells and their components can hinder the recognition and function of macrophages that reside in or enter the skin to clear infections. This disabling of host phagocytosis is undoubtedly central to how *B. dendrobatidis* prevents effective innate and adaptive immune responses in the skin.

## INTRODUCTION

Amphibian populations worldwide continue to decline due to a combination of factors. The most recent assessments of threats to amphibians found that amphibians are the most threatened class of vertebrates, with approximately 40% of species being globally impacted. Causes of these declines include habitat destruction, climate change, and the disease chytridiomycosis (1, 2). Chytridiomycosis was first described in 1998, following severe declines in amphibian populations in Australia and Central America (3). The responsible pathogen was identified, characterized, and named in the following year as *Batrachochytrium dendrobatidis* (4). Current evidence suggests that this pathogen emerged from Asia and spread globally due to amphibian trade (5). The infectious stage of *B. dendrobatidis* is the flagellated naked zoospore (lacking a cell wall). To infect, the zoospore must overcome innate barriers in the skin mucus, including antimicrobial peptides, possible anti-*B*. *dendrobatidis* antibodies, lysozyme, and the antifungal metabolites produced by the community of bacteria that inhabit the mucus (reviewed in reference 6). If the zoospore survives, it forms a germ tube and sends the contents of the zoospore into an intracellular compartment of the skin epidermis (7, 8). When amphibian host cells are compromised, the adaptive immune system should be engaged to begin the process of clearance of the pathogen. However, this process often seems to be ineffective, and the skin integrity continues to deteriorate until the host dies from a lack of uptake of essential ions across the skin (9). The likely first step in detecting these infections is recognition of pathogen-associated molecular patterns (PAMPs) by skin-resident immune populations, chiefly among them resident macrophages (Mϕs) (reviewed in reference 10). In contrast, although there is evidence that amphibian epithelial cell lines can recognize poly (I:C), an analog of viral double-stranded RNA, and respond by the expression of cytokines (11), there is no evidence that they are able to recognize *B. dendrobatidis* PAMPs. The description of pathogenic lesions in the skin notes that inflammation was inconsistent with low numbers of neutrophils, lymphocytes, and few Mϕs (12). We recently showed that at least two subsets of Mϕs (CSF-1-Mϕ and IL-34-Mϕ) are found in the frog skin, playing different roles in anti-*B*. *dendrobatidis* defenses (13). Given that *B. dendrobatidis* can directly impair lymphocyte functions (14) and alter Mϕ functionality (13), we hypothesized that it may also evade immune defenses by targeting the initial recognition and clearance by Mϕs. Many chytrid infections lead to disease without clearance, and thus, we explored the hypothesis that *B. dendrobatidis* interferes with host Mϕ phagocytosis.

## RESULTS

### *B. dendrobatidis* zoosporangia inhibit phagocytic capacities of *Escherichia coli*-elicited peritoneal leukocytes

**Fig 1:**
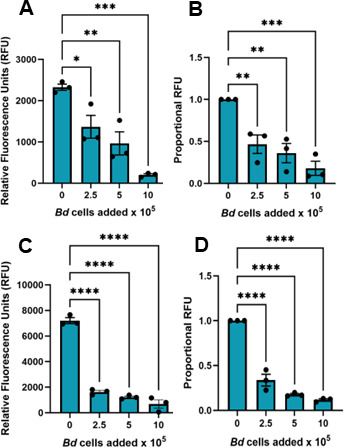
*B. dendrobatidis* zoosporangia inhibit phagocytic capacities of *E. coli*-elicited peritoneal leukocytes. Peritoneal leukocytes (2 × 10^5^/well), induced by injection of heat-killed *E. coli*, were co-cultured with 2-day-old live (**A, B**) or heat-killed (**C, D**) developing zoosporangia for 24 h. Following this incubation, the leukocytes were exposed to pHrodo Green Zymosan BioParticles for 4 h, and uptake of the bioparticles into the acidic phagolysosome was measured as relative fluorescence units (RFU). (**A, C**) One representative experiment of three replicate experiments. (**B, D**) Average fluorescence represented as proportional RFU for three replicate experiments. Maximal RFU in peritoneal leukocytes alone was set at a value of 1. Relative fluoresence (RFU) in the presence of *B. dendrobatidis* cells was significantly different from that of peritoneal leukocytes alone by one-way ANOVA with Dunnett’s *post hoc* test, *P < 0.05, **P < 0.01, ***P < 0.001, and ****P < 0.0001.

**Fig 2:**
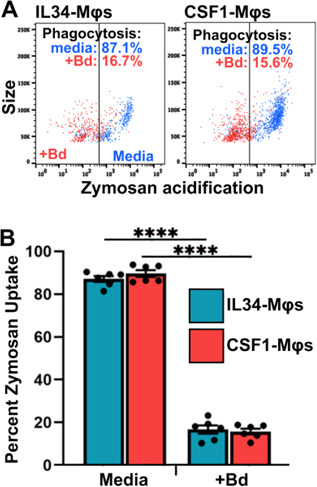
*B. dendrobatidis* zoosporangia inhibit phagocytic capacities of *X. laevis* bone marrow-derived macrophage subsets. Frog bone marrow-derived Mϕs differentiated *in vitro* with recombinant colony-stimulating factor-1 or interleukin-34 were co-cultured with live *B. dendrobatidis* (*Bd*) zoosporangia at a ratio of 1:5 Mϕs to fungal cells for 24 h. Uptake and acidification of zymosan beads were subsequently assessed by flow cytometry. (**A**) Representative scatter plots of pHrodo-positive IL-34- or CSF-1- Mϕs, incubated alone (media) or with *Bd*. (**B**) Percent zymosan uptake in Mϕ subsets generated from 6 individual frogs (*N* = 6). Phagocytosis was significantly inhibited in the presence of *Bd*, ****P < 0.0001 by Welch’s *t*-test.

Enriched macrophages (Mϕs) were induced in the peritoneum of *Xenopus laevis* (MHC-homozygous J-strain) (15, 16) following injection of heat-killed *E. coli*. Phagocytosis by these peritoneal leukocytes (PLs), assessed as uptake and fluorescence of pHrodo Green Zymosan BioParticles, was inhibited after 24-h co-culture with either live (Fig. 1A and B) or heat-killed *B. dendrobatidis* zoosporangia (2-day-old thalli; Fig. 1C and D). Because the PLs contain other leukocyte subsets, including neutrophils and eosinophils, we also examined the phagocytosis of zymosan particles by frog bone marrow-derived Mϕs, differentiated with recombinant forms of principal Mϕ growth factors, colony stimulating-factor-1 (CSF-1) or interleukin-34 (IL-34). Phagocytosis of zymosan particles by these purified bone marrow-derived Mϕs, detected by flow cytometry, was also impaired by co-culture with live *B. dendrobatidis* cells (Fig. 2). This demonstrates that both live or dead *B. dendrobatidis* display or release inhibitory factors to inhibit the functions of peritoneal macrophages as well as bone marrow-derived Mϕs.

### *B. dendrobatidis* zoosporangia and zoospores inhibit phagocytosis of *Staphylococcus aureus* (*S. aureus*) bioparticles by resident peritoneal leukocytes

**Fig 3:**
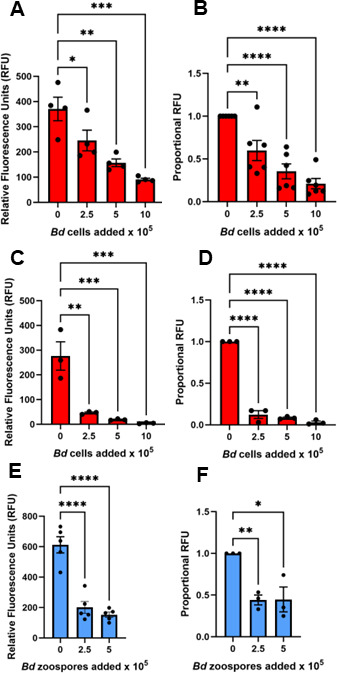
*B. dendrobatidis* zoosporangia and zoospores inhibit phagocytosis of *Staphylococcus aureus (S. aureus*) bioparticles by resident peritoneal leukocytes. Resident peritoneal leukocytes (PLs) (2 × 10^5^/well) were cultured alone or with 2.5, 5, or 10 × 10^5^ 3-day old *B. dendrobatidis (Bd*) zoosporangia/well for 2 h (**A, B**) or 24 h (**C, D**) or with 2.5 or 5 × 10^5^ freshly isolated and freeze-thawed *Bd* zoospores/well for 24 h (**E, F**) at 26°C and incubated for 2 h in the presence of pHrodo Red *S. aureus* BioParticles. Uptake of bioparticles was measured as relative fluorescence units (RFU). (**A**) One representative of six replicate experiments. (**B**) Proportional RFU for six replicate experiments. (**C**) One representative of three replicate experiments. (**D**) Proportional RFU for three replicate experiments. (**E**) One representative of three replicate experiments. (**F**) Proportional RFU for three replicate experiments. Maximal RFU in peritoneal leukocytes alone was set at a value of 1. Significantly different from controls, *P < 0.05, **P < 0.01, ***P < 0.001, and ****P < 0.0001 by one-way ANOVA with Dunnett’s *post hoc* test.

Peritoneal cells elicited by killed bacteria are thought to be pre-activated and may be less representative of macrophages patrolling the skin. Thus, we next examined the effects of *B. dendrobatidis* on phagocytosis by resident (not *E. coli*-elicited) PLs in co-culture with maturing zoosporangia (2-day-old thalli) or immature zoospores. The use of pHrodo Red *S. aureus* BioParticles in addition to zymosan bioparticles was also intended to confirm that the inhibitory factors act generally on phagocytosis, irrespective of the target. Indeed, phagocytosis of pHrodo Red *S. aureus* BioParticles was inhibited as early as 2 h following incubation with developing zoosporangia (Fig. 3A and B) and was further inhibited after 24 h of co-incubation (Fig. 3C and D). Zoospores lack a cell wall, and thus, we tested whether these immature cells would also inhibit phagocytosis. To prevent the zoospores from maturing and developing a cell wall in culture, they were subjected to freeze-thaw cycles to kill them. These inactive zoospores also inhibited phagocytosis by resident (not elicited) PLs (Fig. 3E and F).

### *B. dendrobatidis* cell-free supernatants and cell wall fragments inhibit phagocytosis of *S. aureus* bioparticles by resident peritoneal leukocytes

**Fig 4:**
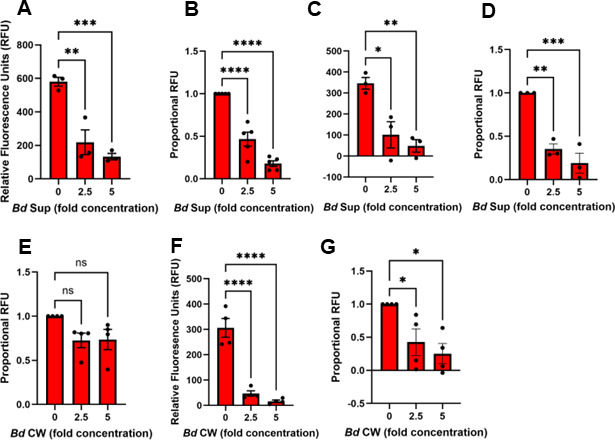
*B. dendrobatidis* cell-free supernatants and cell wall fragments inhibit phagocytosis of *S. aureus* bioparticles by resident peritoneal leukocytes. Resident peritoneal leukocytes (2 × 10^5^/well) were cultured alone or with 2.5- or 5-fold *B. dendrobatidis* supernatant (*Bd* Sup) for 2 h (**A, B**) or 24 h (**C, D**) or with 2.5- or 5-fold *B. dendrobatidis* cell wall fragments (*Bd* CW) for 2 h (**E**) or 24 h (**F, G**) and incubated for 2 h in the presence of pHrodo Red *S. aureus* BioParticles at 26°C. After 2 h, the uptake of red bacteria was measured as relative fluorescence units (RFU). (**A**) One representative of five replicate experiments. (**B**) Proportional RFU for five replicate experiments. (**C**) One representative of three replicate experiments (**D**) Proportional RFU for three replicate experiments. (**E**) Proportional RFU for four replicate experiments of PLs exposed to *Bd* CW for 2 h. (**F**). One representative of four replicate experiments of PLs exposed to *Bd* CW for 24 h. (**G**) Proportional RFU for four replicate experiments of PLs exposed to CW for 24 h. Maximal RFU in peritoneal leukocytes alone was set at a value of 1. Significantly different from controls, *P < 0.05, **P < 0.01, ***P < 0.001, and ****P < 0.0001 by one-way ANOVA with Dunnett’s *post hoc* test.

To determine whether direct contact of Mϕs with *B. dendrobatidis* cells was necessary for inhibition of phagocytosis, we tested the effects of enriched PLs with filtered (0.2 µm) *B. dendrobatidis* cell-free supernatants (see Methods). Like the results with mature *B. dendrobatidis* zoosporangia, the cell-free supernatants (*Bd* Sups) also significantly inhibited phagocytosis after only 2 h of incubation (Fig. 4A and B), and this inhibition was increased after 24 h of treatment (Fig. 4C and D). Because both live and dead *B. dendrobatidis* cells inhibited phagocytosis of zymosan particles, we hypothesized that a factor associated with the cell wall could be a key source of the inhibitory functions. To test whether cell walls would inhibit growth, we adopted a protocol from one used to isolate the cell walls of *Candida albicans* (see Methods). In replicate experiments, the cell wall fragments did not significantly inhibit phagocytosis after 2 h (Fig. 4E, *P* = 0.0745 and 0.0852 for 2.5- and 5-fold cell wall concentrations, respectively), but they did significantly ablate phagocytosis 24 h after exposure (Fig. 4F and G). This suggests that the cell wall-mediated inhibition requires longer exposure to exert its full effect.

### *B. dendrobatidis* zoosporangia, supernatants, and cell wall fragments inhibit phagocytosis of *S. aureus* bioparticles by immortalized murine macrophages

**Fig 5:**
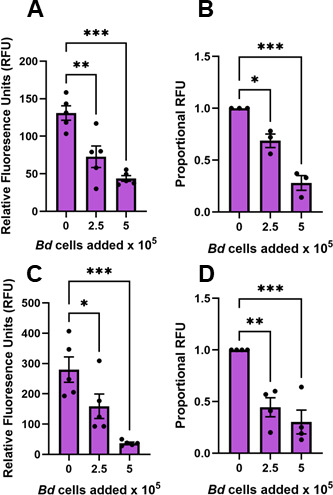
*B. dendrobatidis* zoosporangia inhibit phagocytosis of *S. aureus* bioparticles by immortalized murine macrophages. M0-phenotype macrophages (1 × 10^5^/well) were cultured alone or with 2.5 or 5 × 10^5^ heat-killed *B. dendrobatidis* zoosporangia for 2 h (**A, B**) or 24 h (**C, D**) at 37°C and exposed to pHrodo Red *S. aureus* BioParticles for 2 h. Uptake of bioparticles was measured as relative fluorescence units (RFU). (**A**) One representative of three replicate experiments. (**B**) Proportional RFU for three experiments. (**C**) One representative of four replicate experiments. (**D**) Proportional RFU for four experiments. Maximal RFU in M0 cells alone was set at a value of 1. Significantly different from controls, *P < 0.05, **P < 0.01, and ***P < 0.001 by one-way ANOVA with Dunnett’s *post hoc* test.

**Fig 6:**
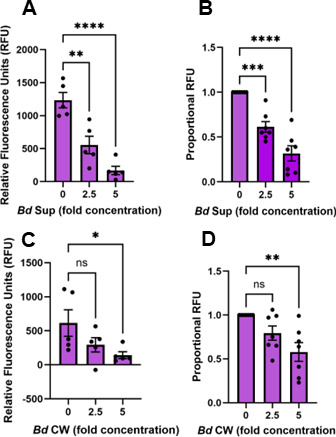
*B. dendrobatidis* supernatants (*Bd* Sups), and cell wall fragments (*Bd* CW) inhibit phagocytosis of *S. aureus* bioparticles by immortalized murine macrophages. M0-phenotype macrophages (1 × 10^5^/well) were cultured alone or with 2.5- or 5-fold *B. dendrobatidis* supernatant (*Bd* Sup) (**A, B**) or cell wall fragments (*Bd* CW) (**C, D**) for 24 h at 37°C and exposed to pHrodo Red *S. aureus* BioParticles for 2 h. Uptake of bioparticles was measured as relative fluorescence units (RFU). (**A**) One representative of seven replicate experiments. (**B**) Proportional RFU for seven experiments. (**C**) One representative of seven replicate experiments. (**D**) Proportional RFU for seven experiments. Maximal RFU in M0 cells alone was set at a value of 1. Significantly different from controls, *P < 0.05, **P < 0.01, and ***P < 0.001 by one-way ANOVA with Dunnett’s *post hoc* test.

To investigate whether the inhibition of macrophage functions by *B. dendrobatidis* was limited to amphibian Mϕs, we examined the effects of *B. dendrobatidis* on phagocytosis by immortalized murine Mϕs. The cell line tested was derived from bone marrow-derived stem cells through retrovirus transformation. When propagated in the standard culture medium, the cell line retains an undifferentiated M0-type phenotype (see Methods). Because *B. dendrobatidis* would be killed by temperatures above 28°C (17), we tested the effects of heat-killed *B. dendrobatidis* zoosporangia in culture with murine macrophages at 37°C. Like the effects of heat-killed *B. dendrobatidis* cells on frog PLs, the phagocytic capacities of immortalized murine Mϕs were significantly inhibited by exposure to heat-killed *B. dendrobatidis* zoosporangia for 2 h (Fig. 5A and B) or 24 h (Fig. 5C and D). To determine whether cell wall-associated factors or other cell-free soluble factors were the dominant source of inhibition of murine Mϕs, we separately tested the effects of filtered (0.2 µm) *B. dendrobatidis* cell-free supernatants (*Bd* Sups) and cell wall fragments (*Bd* CW) on Mϕ phagocytosis. While the supernatants were significantly inhibitory at both 2.5- or 5-fold concentrations (Fig. 6A and B), the cell wall fragments were less inhibitory at a 2.5-fold concentration and required an approximately 5-fold cell wall equivalent concentration (see Methods) to inhibit phagocytosis (Fig. 6C and D).

### Phagocytosis by peritoneal leukocytes and murine macrophages is impaired by *B. dendrobatidis* cells or cell-derived factors with limited changes in cell viability

**Fig 7:**
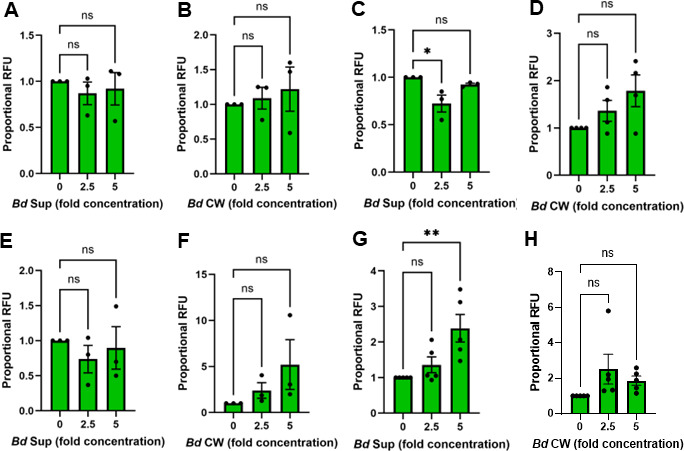
Viability of PLs and BMDM macrophage populations after 2- or 24-h exposure to *B. dendrobatidis* supernatants (*Bd* Sup) or cell wall fragments (*Bd* CW). Resident peritoneal leukocytes (2 × 10^5^/well) were cultured alone or with (**A**) 2.5- or 5-fold *B. dendrobatidis* supernatant (*Bd* Sup) for 2 h or (**B**) 2.5- or 5-fold *B. dendrobatidis* cell wall fragments (*Bd* CW) for 2 h or (**C**) 2.5- or 5-fold *Bd* Sup for 24 h or (**D**) 2.5- or 5-fold *Bd* CW for 24 h. M0-phenotype macrophages (1 × 10^5^/well) were cultured alone or with (**E**) 2.5- or 5-fold *Bd* Sup for 2 h or (**F**) 2.5- or 5-fold *Bd* CW for 2 h or (**G**) 2.5- or 5-fold *Bd* Sup for 24 h or (**H**) 2.5- or 5-fold *Bd* CW for 24 h. After incubation, the cells were stained with SYTOX Green and cell death monitored as nuclear green fluorescence reported as relative fluorescence units (RFU). Results were expressed as proportional RFU for three or more replicate experiments under each set of conditions. Maximal RFU was set at a value of 1. Significantly different from controls, *P < 0.05; **P < 0.01 by one-way ANOVA with Dunnett’s *post hoc* test.

To determine whether loss of fluorescence after treatments was due to increased cell deaths, we assessed viability by both trypan blue exclusion (for murine macrophages) and nuclear staining with SYTOX Green for both PLs and murine macrophages. Viability of PLs determined by SYTOX Green staining after 2-h treatments with *B. dendrobatidis* supernatants or *B. dendrobatidis* cell wall fragments was not different from that of PLs alone (Fig. 7A and B). After 24-h treatments of PLs with *B. dendrobatidis* supernatants or *B. dendrobatidis* cell wall fragments, there was a slight reduction in cell viability after exposure to 2.5-fold supernatant concentration, but not the 5-fold supernatant concentration (Fig. 7C) and no difference due to the cell wall factors (Fig. 5D). Viability of the M0 macrophages determined by trypan blue exclusion after 2 h of treatment was slightly reduced when co-cultured with heat-killed *B. dendrobatidis* zoosporangia to about 85% viability. With all other 2-h treatments (killed zoospores, *B. dendrobatidis supernatants*, and *B. dendrobatidis* cell walls), viability determined by trypan blue exclusion remained very high, averaging 96%–99% viable. With the SYTOX Green assay of murine macrophages, viability after 2-h treatments with *B. dendrobatidis* supernatants or cell wall factors was not different from that of controls (Fig. 7E and F). However, the viability was slightly reduced after treatment with 5-fold *B. dendrobatidis* supernatant after 24 h of exposure (Fig. 7G), but not by the cell wall factors (Fig. 7H).

## DISCUSSION

### Interactions of *B. dendrobatidis* cells and components with frog Mϕs

The interactions between *B. dendrobatidis* fungal cells invading amphibian skin and the skin-resident immune compartment remain poorly understood. Here, we explored frog peritoneal and bone marrow-derived Mϕs as surrogates for frog skin-resident antigen-presenting cells to assess how a key Mϕ functionality (phagocytosis) is impacted by *B. dendrobatidis*. Indeed, our findings indicate that the capacity of *B. dendrobatidis* to inhibit Mϕ phagocytosis extends beyond frog peritoneal-derived cells to bone marrow-derived, growth factor-differentiated CSF1- and IL-34-Mϕs as well as transformed murine bone marrow Mϕs. Peritoneal leukocytes induced by injection of killed *E. coli* are enriched in Mϕs and were significantly impaired in their ability to ingest zymosan particles regardless of whether they were in contact with living *B. dendrobatidis* thalli (2 days of development after zoospore enrichment) or with heat-killed thalli (Fig. 1). Comparing Fig. 1B and D, it appears that dead zoosporangia are as good or better at inhibiting phagocytosis than live thalli. Moreover, resident peritoneal Mϕs were also inhibited by exposure to developing *B. dendrobatidis* thalli for as little as 2 hours, and the effect was more apparent at 24-h exposure (Fig. 3). Comparing Fig. 1 and 3, it appears that resident PLs are equally sensitive to *B. dendrobatidis* zoosporangia as *E. coli*-induced PLs. This suggests that prior macrophage activation is not required for inhibition. Overall, the results show that the factors that inhibit macrophage functions may be released by dying cells, or they are found on the cell walls, or perhaps both soluble factors and cell-wall factors may be involved. This is supported by the effects of cell-free soluble supernatants (*Bd* Sups) as well as fragmented cell walls (*Bd* CW) (Fig. 4). Zoospores lack cell walls, and highly purified zoospores did not inhibit lymphocytes in previous studies (14). Thus, we expected that zoospores would have little or no effect on macrophage function. However, they did inhibit phagocytosis in a dose-dependent manner (Fig. 3C and D). Because the enriched zoospores were only approximately 80% pure and contained a small fraction of developing thalli, the small numbers of maturing thalli may have been sufficient to inhibit the Mϕs.

In mammals, disparate populations of Mϕ-lineage cells reside in and patrol distinct tissues, and the same is thought to be true of amphibians. Mammalian Langerhans cells are the skin-resident immune sentinels (reviewed in reference 18), and Langerhans-like cells have been reported in amphibians (19). Notably, the differentiation and functionality of all vertebrate Mϕs depend on the colony-stimulating factor-1 receptor (CSF-1R), which is ligated by CSF1 and IL-34 cytokines (20). Of these, IL-34 appears to be critical for steady-state Langerhans cell development (21), whereas CSF-1 seems to generate Langerhans cells during inflammatory responses (22). Our recent work indicates that enrichment of *X. laevis* skin IL-34-Mϕs but not CSF1-Mϕs results in anti-*B*. *dendrobatidis* protection *in vivo* (13). Moreover, bone marrow-derived CSF1- and IL-34-Mϕs exhibit markedly different responses to *B. dendrobatidis in vitro,* while both subtypes were able to phagocytose *B. dendrobatidis* zoospores and sporangia (13). We were thus surprised by our present observation that *B. dendrobatidis* suppression of phagocytosis was equally apparent in both frog Mϕ subsets. This supports the idea that *B. dendrobatidis* has evolved a profound capacity to indiscriminately incapacitate Mϕ functionality.

### Interactions of *B. dendrobatidis* cells and components with mammalian Mϕs

Since macrophage functions are evolutionarily conserved (reviewed in reference 23), we also examined the effects of *B. dendrobatidis* cells, supernatants, and cell wall fragments on an immortalized murine cell line. This cell line, which shows characteristics of an M0-type naïve Mϕ, was also suppressed by killed *B. dendrobatidis* cells, cell-free supernatants, and *B. dendrobatidis* cell wall fragments (Fig. 5 and 6). Comparing Fig. 2 and 5D, we observed that 5-fold numbers of *B. dendrobatidis* sporangia inhibited zymosan uptake by *Xenopus* macrophage subsets by about 80%, shown in Fig. 2, while 5-fold numbers of *B. dendrobatidis* zoosporangia inhibited murine macrophage phagocytosis by about 70%, shown in Fig. 5D. Thus, the level of inhibition was comparable. Therefore, we can conclude that the impact of *B. dendrobatidis* fungal factors is not limited to amphibian immune cells. Although the primary relevance of our findings relates to evasion of immunity in amphibians, this immortalized mouse cell line provides another tool to better understand how *B. dendrobatidis* influences Mϕ functions.

### Potential mechanisms of inhibition by *B. dendrobatidis*

Many fungal pathogens are known for their capacities to evade phagocytosis, including inhibiting receptor-mediated phagocytosis (24); evading pattern recognition receptor detection and uptake (25, 26); and even escaping phagolysosomes, once ingested (24). Moreover, fungi can impair the general process of phagocytosis by degrading opsonins on phagocytic targets (27) and releasing extracellular components such as polysaccharides (28) and secondary metabolites (29) that ablate overall phagocytic capacities. In these later examples, the fungi do not simply suppress phagocytosis but seem to “paralyze” the phagocytes, thereby substantially weakening host immune responses. Our findings suggest that *B. dendrobatidis* employs a strategy of incapacitating phagocytosis, an immune mechanism crucial to the success of both innate and adaptive immune responses. Specific mechanisms by which *B. dendrobatidis* may inhibit phagocytosis include interference with actin polymerization, interference with phagosome lysosomal fusion and acidification, and changes in macrophage expression of pathogen recognition receptors, among others. Our previous work indicates that *B. dendrobatidis* elicits substantially increased expression of interleukin-10 (IL-10) by both *B. dendrobatidis*-susceptible and -resistant macrophage subsets (13). IL-10 is widely known for ablating macrophage antimicrobial responses, so undoubtedly *B. dendrobatidis* and *B. dendrobatidis*-derived factors culminate in macrophages inhibiting their own phagocytosis through autocrine IL-10 stimulation. Our previous studies also showed that *B. dendrobatidis* impairs splenic lymphocyte proliferation and causes apoptosis of these cells (14). This current study shows no significant loss of viability of PLs at 2 h of treatment with *B. dendrobatidis* zoosporangia and supernatants when phagocytosis was effectively reduced. Thus, while induction of some cell death may occur after 24 h of treatment with *B. dendrobatidis* supernatant, phagocytosis is impaired well before some death occurred. Ongoing studies will examine additional mechanisms by which macrophage functions are impaired.

## MATERIALS AND METHODS

### Study design

A series of experiments were designed to evaluate the effects of *B. dendrobatidis* cells and cell-derived factors on phagocytosis by enriched populations of amphibian macrophages and an immortalized mouse macrophage cell line.

### Frogs

Inbred *Xenopus laevis* of the MHC homozygous J-strain (originally called the “G Group”) (15, 16), ranging in size from 30 to 100 g, were bred at Vanderbilt University Medical Center and held in polystyrene containers at a density of about 10 frogs per 16 L of de-chlorinated tap water. Frogs were maintained at a temperature between 20°C and 24°C. All animal procedures were approved by the Institutional Animal Care and Use Committee of Vanderbilt University School of Medicine.

### Macrophage sources

Peritoneal leukocytes (PLs) were obtained by lavage without induction or following induction by injection of heat-killed *Escherichia coli* as previously described (14, 30, 31). The dead *E. coli* cells (300 μL, 9.7 × 10^9^ killed CFUs) were injected intraperitoneally into frogs, and 3 days later, the same individuals were anesthetized, and 5 to 10 mL of amphibian phosphate-buffered saline (APBS) (32) were injected into the peritoneum using an 18G × 1 ½” (1.2 mm × 40 mm) needle and a Luer-lock 10 mL syringe. After a gentle massage of the abdomen, the syringe was disconnected, keeping the needle in place, and the volume was drained from the peritoneum into a sterile tube. To obtain uninduced PLs, frogs were anesthetized, injected with APBS, and the fluid was drained as described. For most experiments, the peritoneal fluid was collected in a sterile 50 mL conical tube containing 10 mL of sterile buffered salt solution containing glucose (GKN; 2 g glucose, 0.4 g KCl, 8 g NaCl, 2.68 g Na₂HPO₄•7H₂O, 0.78 g NaH₂PO₄•2H₂O, and 0.01 g phenol red per liter of glass-distilled water, adjusted to pH 7.2). To obtain sufficient cells, cells from two or more MHC homozygous J-strain frogs were pooled.

*Xenopus* bone marrow-derived macrophage subsets were obtained by recombinant cytokine-induced outgrowth of bone marrow stem cells, as previously described (33). Recombinant interleukin-34 (rIL-34) and recombinant colony-stimulating factor-1 (rCSF-1) were produced as previously described (13). Briefly, signal-peptide-cleaved transcripts corresponding to *X. laevis* CSF-1 or IL-34 were PCR-amplified and ligated into the pMIB/V5 His A insect expression vector (Invitrogen, Waltham, MA, USA) and used to transfect Sf9 insect cells. Western blot analysis against the V5 epitope was performed on the supernatants from transfected Sf9 cells to confirm the expression of rCSF-1 or rIL-34, following selection of positive transfectants using 10 μg/mL blasticidin.

Immortalized bone marrow-derived murine macrophages (BMDM) were derived in the Serezani laboratory (34). Briefly, bone marrow cells from 10-week-old C57BL/6 mice were collected and stimulated to proliferate with 50 units/mL granulocyte macrophage-colony stimulating factor (GM-CSF) and 5–10 ng/mL of macrophage colony-stimulating factor (M-CSF). After outgrowth, the cells were infected with a suspension of J2 retroviruses. After infections, clusters of non-adherent rapidly proliferating cells were dissociated and cloned by limiting dilution in Dulbecco’s modified Eagle medium (DMEM) supplemented with 10% fetal bovine serum. The cell line was maintained by splitting 1:10 twice weekly in DMEM with 10% fetal bovine serum and 100 IU/mL penicillin and 100 mg/mL streptomycin.

### Experimental design and phagocytosis assays

Peritoneal leukocytes (PLs) were collected as described above and centrifuged at 600 × *g* for 10 min to pellet them. The supernatant was discarded, and the pellet was resuspended in 1 mL of Leibovitz-15 (L-15) medium, which had been adjusted to amphibian osmolarity and supplemented with 100 IU/mL penicillin, 100 mg/mL streptomycin, 12.5 mM sodium bicarbonate, 50 mM 2-mercaptoethanol, 2 mM L-glutamine, and 1% heat-inactivated fetal calf serum. A small aliquot was removed, diluted in 0.4% trypan blue dissolved in APBS, and counted using a hemocytometer slide. After counting, the cells were adjusted to a concentration of 2 × 10⁶ cells/mL, and 200,000 PLs/well were plated in 96-well black-sided, clear, flat-bottom tissue culture-treated plates (Corning #3603, Glendale, AZ, USA). For most experiments, the cells were allowed to adhere for 2 h at 26°C to enrich macrophages, and then the wells were washed twice with APBS to remove any non-adherent cells, leaving about 100,000 adherent macrophages per well.

Adherent BMDM cells were dislodged by treatment with 0.25% (wt/vol) trypsin-EDTA and diluted to 2 × 10⁶ cells/mL for plating at 50 µL (100,000 cells)/well. PLs or BMDM cells were exposed to *B. dendrobatidis* cells or cell-derived factors for 2 or 24 h and incubated at 26°C for PLs or 37°C for BMDM. On the same day for 2-h treatments or the following day for 24-h treatments, 5 µL of pHrodo *S. aureus* red BioParticles or pHrodo Green Zymosan BioParticles (Invitrogen, Waltham, MA, USA) at a concentration of 1 mg/mL were added to each well for 2 or 4 h (see figure legends). Wells were then washed with APBS for amphibian cells or PBS for BMDM, and 100 µL of the appropriate phosphate-buffered saline was added per well before reading fluorescence on a Biotek Synergy LX or a Biotek Cytation 5 plate reader (Agilent, Santa Clara, CA, USA) to quantify phagocytosis of bioparticles within the acidic phagolysosome. For some experiments, images of the cells with or without added bioparticles were acquired at 20× magnification using an overlay of bright-field and Texas Red filters at Ex/Em 560/10-585/10, as per the manufacturer’s recommendation.

### *Batrachochytrium dendrobatidis* cells, supernatants, and cell wall preparations

*B. dendrobatidis* isolate JEL197 was cultured and maintained in 1% tryptone broth (T-broth) at 21°C and sub-cultured twice weekly by splitting the culture 1:10. Zoospores (approximately 80% purity) were collected as previously described (35) by twice flooding 4- to 7-day-old cultures of *B. dendrobatidis* cultured on 1% tryptone agar (10 g tryptone, 10 g agar, and 1,000 mL glass-distilled water) using 3–5 mL of T-broth. The collected zoospores were filtered over sterile nylon spectra/mesh filters (Spectrum Laboratories, Rancho Dominguez, CA, USA) of 20 μm mesh opening to remove mature cells. Most experiments used live zoosporangia developing for 2 days from purified zoospores and then cultured with macrophages for 2 or 24 additional h. Some other experiments used mixed cells from growing liquid cultures, including zoospores, but counted only the larger, more mature cells. For the addition of dead *B. dendrobatidis* to macrophage cultures, the zoosporangia or zoospores were killed by incubation in a water bath at 60°C for 10 or more min.

Supernatants were obtained as previously described (14). Briefly, about 150 mL of actively growing *B. dendrobatidis* cells (containing zoospores and mature zoosporangia) were expanded for 4–5 days in T-broth, counted, and diluted to a concentration of 10^6^/mL in T-broth and cultured for an additional 7 days at 21°C. All cells were then collected in several 50 mL sterile tubes, spun at 2,095 × *g* for 10 min, and the tryptone broth was removed. The cells were washed twice with sterile glass-distilled water. All cells were pooled, counted, and resuspended at a concentration of 10^7^ mature cells/mL (all cells of a greater size than zoospores) in fresh sterile glass-distilled water for 24 h. The cells were then pelleted by centrifugation at 2,095 × *g* for 10 min at 21°C. The supernatant was collected, filter-sterilized, frozen at −20°C, and lyophilized. The lyophilized material was resuspended in L15 or DMEM at 1/10th the original volume of cells that were incubated in the final step at 10^7^/mL to create a 10-fold concentration that was diluted to achieve a 5-fold, 2.5-fold, or 1.25-fold concentration in the experimental wells.

*B. dendrobatidis* cell wall fragments were prepared following the method of Chauhan, (36). The final preparation was resuspended in a volume such that 1 mL contained the cell wall material derived from 10^7^ mature *B. dendrobatidis* zoosporangia. Briefly, about 200 mL of *B. dendrobatidis* cells (all life stages) were grown for 7 days at 21°C in 1% T-broth. Cells were centrifuged at 850 × *g* for 20 min, the supernatant was aspirated, and the cell pellet was resuspended in 5 mL sterile ice-cold glass distilled water for counting. After counting mature cells, the cells were diluted to about 2 × 10⁸ total cells in 5 mL of ice-cold glass distilled water and centrifuged again at 850 × g for 20 min. The resulting cell pellet was washed 5 times with 3 mL ice-cold lysis buffer (10 mM Tris-HCl, pH 7.5) before being resuspended in 5 mL of the same buffer. The cell mixture was then divided evenly into sterile microcentrifuge tubes containing 110 mg of sterile glass beads (0.5 mm zirconia/silica). The mixture was centrifuged for 3 min at 850 × *g*, and the supernatant was removed, leaving about 300 µL/tube. Tubes were then placed on a bead beater at 4.5 m/s for 1 min. This was repeated 12 times. Cell breakage was confirmed under the microscope using a hemocytometer. Cell fragments were pooled into a single tube, centrifuged at 850 × *g* for 10 minutes, and washed in decreasing concentrations of 3 mL NaCl (5%, 2%, and 1% wt/vol in cold distilled water) before washing 5 times in 3 mL ice-cold and sterile glass-distilled water. The final cell pellet was resuspended in 10 mL of cold sterile distilled water, separated into two 5 mL aliquots, frozen at −20°C, and lyophilized. The lyophilized product was resuspended in 10 mL of L15 or DMEM to achieve 1 × 10⁷ cell wall equivalents/mL. Before plating, an 18-gauge needle attached to a 10 mL Luer-lock syringe was used to break up any remaining clusters of cell wall material to ensure the cell wall components were evenly distributed in the culture medium.

### Cell viability assays

To quantify cell viability, adherent BMDM cells were dislodged and plated in the manner described above. PLs or BMDM cells were treated with *B. dendrobatidis* cell-free supernatants or cell wall preparations for 2 or 24 h as previously described and incubated at 26°C for PLs or 37°C for BMDM. Following 2-h or 24-h exposure, 100 µL of 1 µM SYTOX Green Nucleic Acid Stain (Invitrogen, Waltham, MA, USA) diluted in phosphate-free Ringer’s Solution (6.6 g NaCl, 0.15 g KCl, 0.15 g CaCl_2_, and 0.2 g NaHCO_3_/L of glass distilled water) for PLs or phosphate-free Hanks balanced salt solution (HBSS) for BMDM was added to each well and allowed to stain in the dark for 15 min. Wells were then read for fluorescence on a Biotek Synergy LX or a Biotek Cytation 5 plate reader (Agilent, Santa Clara, CA, USA) to quantify the relative number of cells with permeable membranes characteristic of dead cells. For some experiments, images of the cells with or without added nucleic acid stain were acquired at 20× magnification using an overlay of bright-field and GFP filters at Ex/Em 560/10-585/10, as per the manufacturer’s recommendation.

### Statistical analysis

Statistical analyses were performed using GraphPad Prism 10.0 software. Data sets were assessed by one-way ANOVA followed by Dunnett’s *post hoc* tests for multiple comparisons or by Welch’s *t*-test. *P*-values ≤ 0.05 were considered to be statistically significant.

## Data Availability

All data needed to understand these results are summarized in the figures and figure legends. Further details about the data are available upon request.
